# A review of promoting access to medicines in China - problems and recommendations

**DOI:** 10.1186/s12913-018-2875-6

**Published:** 2018-02-20

**Authors:** Jing Sun, Cecile Jia Hu, Mark Stuntz, Hans Hogerzeil, Yuanli Liu

**Affiliations:** 1 School of Public Health, Chinese Academy of Medical Sciences & Peking Union Medical College, 5 Dongdansantiao, Dongcheng District, Beijing 100730 People’s Republic of China; 2 Deerfield Institute, K Wah Center 3703, Middle Huaihai Rd 1010, 200031 Shanghai, People’s Republic of China; 3 Deerfield Institute, 780 Third Avenue, 37th Floor, New York, NY USA; 4 Department of Health Sciences, University Medical Centre Groningen, University of Groningen, Groningen, 9700 RB The Netherlands

**Keywords:** China, Review, Access to medicines, Healthcare reforms, Policy recommendations

## Abstract

**Background:**

Despite recent reforms, distorting funding mechanisms and over-prescribing still maintain severe financial barriers to medicines access in China. Complicated and interrelated problems in the pharmaceutical sector require a common framework to be resolved as fragmented solutions do not work. We present a preliminary assessment of the impact of the national healthcare reforms on access to medicines, and propose policy recommendations for promoting universal access to medicines in China.

**Methods:**

Drawing on multiple sources of information, including a review of published literatures and official national data, field investigations in six provinces and interviews with key opinion leaders, this paper presents a preliminary assessment of the impact of the national healthcare reforms on access to medicines, and proposes policy recommendations for promoting universal access to medicines in China.

**Results:**

Public expenditure on medicines has been strictly controlled since the national healthcare reforms of 2009. Yet total pharmaceutical expenditure (TPE) and total health expenditure growth rates continuously outpaced the growth of gross domestic product (GDP). With 2.4% of GDP, TPE now exceeds that of most high income countries. The distorted provider and consumer incentives in the Chinese health system have not fundamentally changed. Price-setting and reimbursement mechanisms do not promote cost-effective use of medicines. Inappropriate price controls and perverse financial incentives are the un-resolved root causes of preference of originator brands for some major diseases and shortages of low-cost and low-consumption medicines. In addition, access to expensive life-saving medicines is yet systematically addressed.

**Conclusions:**

The complicated and interdependent problems interact in a way that leads to significant system problems in China, which create dual challenges that both the developing country and the developed countries are facing. To further promote access to medicines, China should speed up the re-assessment of the quality and efficacy of domestically produced generic medicines; coordinate various reforms of price determination, insurance payments, and procurement policies; address medicine shortages through comprehensive policies and legislation; establish specific mechanisms to achieve sustainable equitable access to expensive essential medicines with health technology assessment as a tool to ensure that policy and priority setting are created in a coherent and evidence-based way.

**Electronic supplementary material:**

The online version of this article (10.1186/s12913-018-2875-6) contains supplementary material, which is available to authorized users.

## Background

Since the early 1980s, China’s economy has been growing at approximately 10% per year [1]. The living standard and the healthcare needs of the Chinese people have been continuously increasing. Similarly, the Chinese pharmaceutical industry has been rapidly growing at an annual rate of 15–25% (gross output value) since 1978 [2]. However, the health outcomes of the Chinese people between 1980 and 2010 did not improve at a commensurate rate as that of the general economy development during the same time period. This is even lower than many countries which were at the same economic development level like China in the 1980s [3]. The 2nd National Health Service Survey conducted in 1998 found that the poverty rate caused by illness in rural areas was 21.61%, and could reach 50% in poor rural areas [4]. The Development and Research Center of the State Council published a report in 2005 together with the World Health Organization (WHO), which concluded that the rapid increase of healthcare expenditures brought heavy financial burden to the individuals, and subsequently led to financial barriers for access to medicines. One of the key reasons behind this is the out-of control prices of medicine and over-prescribing of medicines [5]. Since 2009,there has been a national health system reform implemented in China, reforms in medicines area are innumerable. Despite recent reforms, distorting funding mechanisms and over-prescribing maintain severe financial barriers to medicines access. This review is committed by the National Health and Family Planning Commission of P.R. China in 2016. The analysis of the key reform policies and problems in the Chinese pharmaceutical sector aims to help the international readership to better understand the essence and limitations of various major reform initiatives, to contribute better promotion of sustainable equal access to and cost-effective use of quality medicines in China, and maybe serve as a reference for other settings.

## Methods

A multi-faceted approach incorporating various data sources was used for this study. Firstly, we listed the major pharmaceutical sector reform policies issued by the central government since 1997. Secondly, as over-prescribing is one outstanding characteristic of the Chinese pharmaceutical sector [5], we looked at the trends of medicines expenditures at both macro (1990–2015) and micro levels (2008–2015) based on official national statistics. Thirdly, a systematic review of grey and published literatures was followed. Chinese government documents (National Reform and Development Committee, National Health and Family Planning Commission, China Food and Drug Administration) were searched for government documents of relevant reform initiatives (Additional file 1). The major Chinese literature databases (CNKI, VIP and Wanfang) and English literature database (MEDLINE) were searched for published literatures about the key reform initiatives issued by the Chinese government. In addition, a series of field investigations were conducted in seven provinces (Shaanxi, Qinghai, Gansu, Hubei, Sichuan, Guangxi and Fujian) to look into policy implementations. Interviews with key opinion leaders and focused group discussions were organized at central, local and global levels to explore underlying issues. An outline of the key informants interviews and focused group discussions were presented in Additional file 2. Core problems relating to access to medicines were analyzed and policy recommendations were proposed, based on the analysis.

## Results

China has a complex and dynamic pharmaceutical system undergoing significant reforms as part of the national health care reforms. Major pharmaceutical sector reform initiatives include, containment of the rapid increase of pharmaceutical expenditures; formulation of appropriate medicines financing mechanisms including pricing of medicines, medical services, medical staff salary scales, reimbursement policies, and financing of public hospitals; secure stable and efficient supply and procurement of medicines; and secure sustainable universal access to essential medicines. The complicated and interdependent problems interact in a way that leads to significant system problems in the Chinese pharmaceutical sector. China has facing dual challenges that both the developing country and the developed countries are facing, i.e. quality and affordability of medicines, common essential medicines are increasingly missing and replaced with high-priced alternatives, rapid rise of medicines expenditures and sustainable mechanisms of access to expensive medicines after achieving universal coverage of basic healthcare. Unilateral policy interventions without a common policy framework did not fundamentally resolve the system problems.

### Pharmaceutical expenditure growth at the macro level

**Fig. 1:**
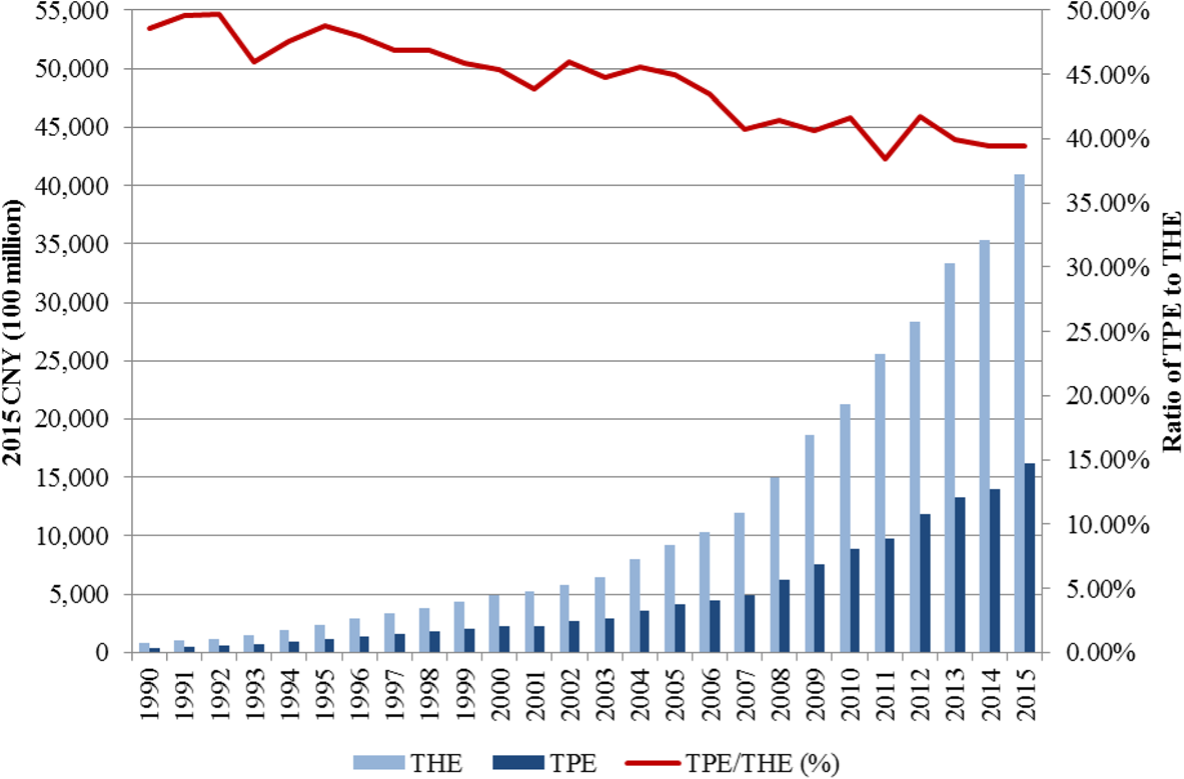
Total Pharmaceutical Expenditure (TPE) and Total Health Expenditure (THE), ratio of TPE to THE (1990–2015)

According to China Health Account Report [6], between 1990 and 2015, the total pharmaceutical expenditure (TPE) continuously grew along with the total health expenditure (THE). The growth rates of both the THE and TPE were generally higher than the growth rate of the gross domestic product (GDP), although the ratio of TPE to THE declined from approximately 50% in 1990 to 39% in 2015 (Fig. 1). This is most likely due to the growth in non-pharmaceutical expenditures (salaries, diagnostics, and hospital beds). The ratio of TPE to GDP has increased to nearly 2.4% – exceeding the top ten Organization for Economic Co-operation and Development member countries with the highest expenditures on medicines as a proportion of GDP [7].

### Pharmaceutical expenditure growth at micro level

**Fig. 2:**
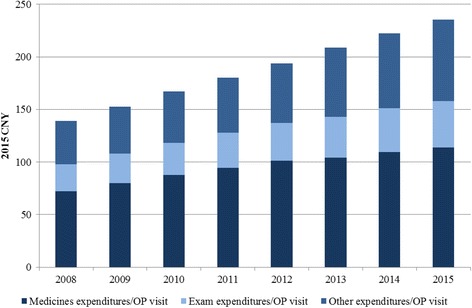
Average expenditures per outpatient visit in Chinese public hospitals (2008–2015)

**Fig. 3:**
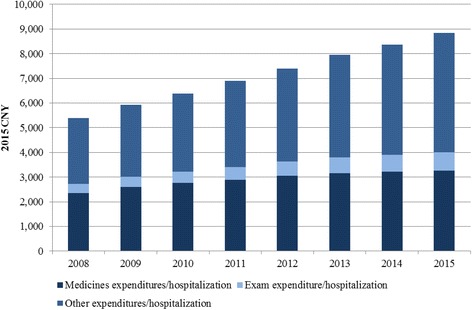
Average expenditures per hospitalization in Chinese public hospitals (2008—2015)

Medicines expenditures in public hospitals have been strictly controlled since the most recent round of national health system reforms in China in 2009. Policies like price-cutting, zero mark-up (public health facilities dispense medicines at the procurement price), and dual invoicing (issuing of the manufacturer’s invoice to the distributor followed by the distributor’s invoice to hospital) all aim to contain the rapid growth of medicines expenditures. However, although the medicines expenditures stayed quite stable, the average expenditure per outpatient visit and per hospitalization in Chinese public hospitals each nearly doubled between 2008 and 2015 (Figs. 2 and 3) [8]. To account for inflation, expenditures data in each year were adjusted to 2015 CNY value using the national consumer price index data [9].

## Discussion

### Key problems and causes

#### Lack of appropriate financing mechanisms for cost-effective use

The pricing policies for medicines and medical services in China are distorted. The government has been expected to control the public medical service prices (including formal salary scales of the medical staff) at low levels in order to secure affordability of healthcare for the public [10]. Unfortunately, medicines have become a source of funding for the public hospitals and a source of informal income for doctors [11]. Before 2015, the prices of medicines were set by the government in China, with many rounds of price-cuttings conducted targeting generics. Numerous generics suppliers encountered fierce price competition during the pooled procurement which tended to force medicines prices down while brand originators enjoyed favored price-setting policy, and many maintained price monopolies even when there was generic competition. The distorted pricing policies have been the driving force of the abnormal preference of high-priced medicines in public hospitals. Low-cost medicines and low-consumption medicines were not attractive. Such phenomenon drove national production towards higher-price medicines [12–14]. After the abolishment of government price-setting policy in 2015, the distortion has been somewhat reversed through implementing market-based prices for medicines. However, encumbered by the poor quality and efficacy of local generics, brand originators are still put into a separate competing category, which continues helping the maintenance of a virtual price monopoly of the brand originators. The new policy of determining medicines price by insurance programs may play a role in breaking this strange loop, which intends to create financial incentives for public hospitals to procure and prescribe the most cost-effective medicines and treatment. This policy is still in the pilot phase, and requires cooperative supporting policies, including the pricing of medical services, salary scales of medical staff, better provider and consumer payment methods, and secured quality and efficacy of generics [15].

#### Medicines shortages are not systematically and strategically addressed

Low-cost medicines were missing from the Chinese medicines market. There have also been an increasing number of shortages of low-consumption medicines, including orphan medicines, and the markets of some products have been dominated by brand originators. An investigation report about medicines shortages in 2010 showed that there were 284 medicines out of stock in different levels of health care facilities, 83% of which were on the National Essential Medicines List, National Reimbursement List of the Basic Health Insurance Program, or the Medicines List for Primary Care [16]. This situation was not improved after 2009 when the national health system reform started. Another investigation report of 2015 indicated that among a list of 780 medicines which were investigated, 357 were totally out of stock [17]. Shortages of pediatrics medicines are also a serious concern. Among the 3500 total marketed medicines, only 60 were pediatric medicines (1.7%) – 90% of medicines do not have pediatric formulations. Of the 6414 pharmaceutical manufacturers, only 30 produce pediatrics medicines [18]. Shortages of pediatrics medicines and pediatric formulations have brought tremendous safety risks. According to the 2015 National Drug Adverse Reaction Monitoring Report, 9·9% of drug adverse reaction events were for children under 14 years old [19].

A report of an online medicines shortages survey in 2016 disclosed that among 8670 responding prescribers, 77% had ever encountered medicines shortage; 53% thought the shortage was due to a smaller number of patients, thereby low prices and low profits of the suppliers; and 30% thought it was due to the procurement policy [20]. Key reasons of medicines shortages in China as: The key reasons of medicines shortages in China were summarized in Table 1.

**Table 1:** The key reasons of medicines shortages in China

Demand-side reasons		Sudden increase of medicines demand may lead to shortage. For the case of some rare disease, the routine quantity of demand is small. Production and transportation of larger quantity within a short time is difficult when there is a sudden increased number of patients under specific conditions. Pressure on inventory control brought by dramatic demand-side change may lead to temporary shortages. Such shortages are always accompanied by a revision of treatment guidelines. If the buffering and alert capacity of the inventory control system is not strong enough, the poor flow of information, finances, and logistics may cause short supply [21].
Supply-side reasons	Regulatory shortages	Shortages may happen when manufacturers temporarily stop production to test the newly revised specifications, or to validate compliance of Good Manufacturing Practice as required by the regulatory authority [22]. In 2007, all Chinese blood products manufacturers were required by the national drug regulatory authority to update the specifications, and to implement the lot release policy. These requirements caused a sharp reduction in the quantity of marketed blood products [23]. The shortage of protamine sulfate in 2011 was due to the fact that manufacturers had to validate the stability of the products after a change of raw materials. This was followed by a similar situation in 2016, when the national pharmacopeia was updated and the manufacturers recalled relevant lots of the marketed products [24].
	Policy shortages	The distorted pricing of medicines, medical services, compensation of medical staff, reimbursement policies, and financing of public hospitals create severe perverse financial incentives in the Chinese health system. These have not been fundamentally changed through the major reform initiatives since 2009, which include price-cuttings and zero mark-up on medicines. Under a chaotic medicines distribution system with irregular finance practices and disordered competitions, the un-ethical relationship between pharmaceutical industry and doctors is still quite common in Chinese public hospitals. The informal payment to public hospitals and doctors continues. As both the demand-side and the supply-side do not have incentives to lower the medicines prices, low-cost medicines do not have market value. Manufacturers tend to produce medicines with higher price and better sales performance. This lead to shortages of those old low-cost medicines. The tough price-cutting may also lead to bid suppression – manufacturers tend to pool the small orders into big ones to pursue the economy of scale, potentially leading to temporary shortages.
	Unfair competition	A few companies monopolize the production of key raw materials for the manufacture of some medicines. Hoarding key raw materials for speculation at low price reduces or ceases the supply, forcing the downstream manufacturers to stop production of pharmaceutical formulations. This leads to fear and panic of downstream manufacturers, allowing the speculators to raise price for illicit profits [25].

#### Lack of sustainable mechanisms to promote universal access to expensive life-saving medicines

With the fast development of biotechnology, some expensive medicines have brought new hope of life for the patients who were formerly sentenced to death. Most high-income countries have formulated public-funded mechanisms to secure universal access to the expensive medicines. However, this issue has just recently been put on the agenda of the Chinese government, and China still equates essential medicines as cheap medicines for common diseases. There is no national legislative foundation for the government to secure equal access to expensive life-saving medicines in China. Although the recent national-level price negotiations produced shocking over 50% cuts to procurement prices for three high-cost medicines in 2016 and 36 in 2017 [26, 27]. There is still a long way to go to achieve effective price negotiation at both central and local levels. There is an urgent need to address this issue strategically, to establish evidence-based inclusion mechanisms for the public-funded access programs, and to secure sustainable financing and supply systems for the high-cost medicines.

### Recommendations to further promote access to medicines in China

#### Ultimate goal: A systematic approach to promoting medicine access

As China is in the process of formulating the National Health Law, it is necessary to stipulate the immediate and long-term national objectives and development priorities of the pharmaceutical sector, and allow the establishment of a multi-ministerial committee in the National Health Law. It is also essential for the National Health Law to authorize the Committee to coordinate issues related to research and development, manufacturing, distribution, utilization, and regulation. It is also imperative to create appropriate incentives for each player in the medicines supply chain with a sustainable long-term development.

#### Specific policies: Mechanisms development

##### Mechanism 1: Speeding up the re-evaluation of quality and clinical efficacy of generics

Many reforms in the pharmaceutical sector including the efforts of containing the medicines expenditure and promoting cost-effective use have a prerequisite that, the quality and clinical efficacy of generics are secured. The ongoing initiative of the national drug regulatory authority to re-evaluate the quality and clinical efficacy of generics is a critical effort to make up for the defectiveness of the drug evaluation system before 2007. The re-evaluation should be sped up and secured to be accomplished before the deadline of the end of 2018 set by the State Council [28]. The regulatory authority needs to strengthen the review team, coordinate with relevant authorities, resolve the problems of appropriate Reference Listed Drug and clinical trial sites, and share the re-evaluation results with openness and transparency [29].

##### Mechanism 2: Coordinated reforms of pricing, insurance payment, and procurement policies

*Coordinated approach:* Successful pharmaceutical policies have to address both the socioeconomic and cultural factors, and adapt to specific settings with a coordinated strategy package [30]. Coordinated pricing policies, insurance payment methods, and medicines procurement policies are the keys for reversing the distorted financial incentives in the Chinese health system. Multiple stakeholders in all relevant systems must collaborate to contain medicines expenditures and promote cost-effective use of medicines.

*Proactive role of health insurance:* Health insurance programs have great potential to improve the cost-effective use of medicines by leveraging better provider prescribing, more cost-effective use by consumers, and lower prices from pharmaceutical companies [31]. Many health insurance systems determine reference prices to be reimbursed for medicines, which is a flat price per item using the International Non-proprietary Name. Reference prices allow the pharmacists or dispensing doctors to keep the profits from any discounts they achieve by sourcing lower-priced medicines from competing wholesalers or direct-to-pharmacy distributers. This provides a profit-based incentive for wholesalers and pharmacists to search for the lowest-cost suppliers [32, 33]. This imparts the strategy that, as the large purchasers, China’s basic health insurance programs could use their monopsony power to exert downward pressure on prices [34].

Considering that China has already achieved universal coverage of health insurance, insurance funds have been the key source of financing of the dominated provider-public hospitals. After the government medicines price-setting policy was abolished in 2015, there has yet to be a medicines price regulation system established. It is time for the health insurance programs to play a role in setting the maximum reimbursement prices in a systematic way – such as including reference prices to determine insurance reimbursement price, aiming to create incentives for cost-effective prescribing and use, and helping to contain health expenditures.

*Appropriate medicines pricing:* For multi-source products (including quality and clinical efficacy secured generics and off-patent originators), internal reference pricing should be used to encourage competition. It is recommended to use lower percentile price as the payment price and leave the providers to bear the risk of excessiveness and keep the surplus to encourage cost-effective prescribing [35]. For the single-source products, comparative cost-effective analysis should be conducted between the innovative medicines and the existing products with the same indication, and those which don’t gain added value should be put into the same category of multi-source products for competition. Only those that have added value should be considered in the non-competitive category. Before the pharmaco-economic assessment capacity and system is well established, external reference pricing is more feasible for China to provide quick information and to determine the best price of the non-competitive medicines [36].

In addition to reform the pricing policy for medicines, increasing prices for the under-valued labor intensive medical services will play an important role in achieving the strategy of fleeing the medicines revenue, so as to filling the medical service revenue, and reversing the distorted pricing and financing systems of public hospitals. Considering its financial impacts on the health insurance fund, the individual financial burden and the operation of public hospitals, the proposed medical service price adjustment should be a dynamic adjustment mechanism based on the change of the cost and revenue structures. The impacts of the price adjustment on relevant stakeholders should be evaluated before and after the adjustment, and the adjustment should be made at appropriate opportunity, with proper range, and within a reasonable time period.

*Provider and patient payment reform:* In order to achieve the optimized motivation objective of the pricing policies, provider payment reform is also a critical strategy. The current resource-exhausted retrospective fee for service payment should be shifted to a mixed prospective payment method, including capitation payments for primary care and case-based payments for specialist care. Such reform tends to motivate providers to deliver the most cost-effective care. Consumer payment should also be shifted from fixed percentage reimbursement to tiered co-payment with generic substitution policy (full reimbursement for generics and lower proportion of reimbursement for non-preferred brand products), in order to encourage cost-effective use of medicines of consumers [37, 38].

*Procurement policy:* The reference price determined by the health insurance programs is a result of multiple gaming among different stakeholders. After the abolishment of government price-setting policy in 2015, medicines prices have been determined by market competition, although there are still government controls over the daily treatment of low-cost medicines. Theoretically, medicines procurement price should be the result of the market competition and an important basis for the health insurance programs to determine the medicines reimbursement price to be paid, although there are still many unreasonable price controls in the process of the government-dominated pool provincial procurement. The procurement price is a valuable reference for the health insurance programs to determine the reference price for payment and price adjustment periodically. Thus, the transparent provincial medicines procurement platform will play an essential role in collecting price and quantity information, and docking with the health insurance programs [39].

##### Mechanism 3: Addressing medicines shortages strategically

*Appropriate price control:* Many cases of medicines shortages in China are due to inappropriate price control. The Lancet Commission on Essential Medicines recommended that affordable prices for medicines be compatible with the sustainability of the pharmaceutical industry [40]. A price regulation mechanism should be feasible and affordable in relation to the technical capacities and resources of the country. Other aspects to be considered are whether the mechanism is objective, non-discretional, predictable, and transparent, as these qualities reduce uncertainty to the suppliers. This potentially leads to lower supply prices and fewer delays in marketing a product. Medicines pricing policy in China should not blindly over-depress the prices, but leave reasonable profit margin for all essential players over the supply chain, including manufacturers, wholesalers, retailers, and logistics supports. Tenders should be awarded to more than one supplier to secure supply. The daily defined fixed price limits should be replaced with ad hoc monitoring of illegal and unethical practices over the supply chain. Incentives to procure and to prescribe cost-effective medicines by public hospitals should also be set up when reforming the provider payment method.

*Enhanced health legislation:* To address medicines shortages strategically, the government should define the responsibilities, institutionalizations and reserve mechanisms legislation. These should be incorporated into the Chinese Drug Administration Law. The securement of the supply of medicines for rare diseases (including pediatrics) should also be defined in the Law.

*Cooperative mechanisms:* One common experience of countries who successfully dealt with medicines shortages is setting multiple cooperative mechanisms along the full drug supply chain, including drug regulatory authorities at all levels, industries, and patients. In addition, developing guidelines for relevant stakeholders, monitoring, analyzing, alerting, announcing and updating shortages, production and distribution quantities, and sharing information across regulatory authorities, industries, health providers and patients are also critical for effective and efficient responsiveness to medicines shortages. Lastly, the national anti- illicit competition and anti-monopoly department should avoid manipulated panic and deliberate shortages.

##### Mechanism 4: Establishing sustainable mechanism for equal access to expensive life-saving medicines

In 2015, WHO included ground-breaking innovative medicines that show clear clinical benefits into its Model List of Essential Medicines. This move opened the way to improve access to expensive medicines and had enormous public health impact globally. Increasingly, governments and institutions around the world are using the WHO list to guide the development of their own essential medicines lists [41]. The Chinese government should response actively to the call of WHO to include cost-effective expensive life-saving medicines into the National Essential Medicines List, and followed with comprehensive supporting policies to promote access to these medicines through setting a special category of essential medicines, evidence-based priority-setting mechanisms, sustainable financing and supply systems, and good government coordination with necessary intellectual property right policy tools.

##### Mechanism 5: Promoting health technology assessment (HTA) to foster evidence-based priority setting

Prices for new medicines are typically set by the manufacturer in order to maximize profit during a period of monopoly supply. One approach to prioritize the most cost-effective innovative medicines is the use of HTA, which refers to the the systematic evaluation of properties, effects, and/or impacts of health technology. Results from HTA have been used by government agencies as an input in price negotiations over new medicines in many OECD countries, and this assessment has been rapidly developing in some middle-income countries [42]. HTA should be legitimized in the decision-making process and backed by high-level policy. Coinciding with the occasion of the national health legislation in China, establishment of a technical body and guideline for conducting HTA should be incorporated into the National Health Law in order to link HTA directly with public health resource allocation, as well as to generate political demand and need for more and better quality HTA. The technical body should be independent of government, refuse financial support from private sources, and have a clear or explicit code of conduct to deal with conflicts of interest. The government should make financial resources available to support HTA so that HTA researchers do not have to rely on industry support. The costs of HTA can be paid from savings on the medicines expenditures.

HTA is a highly analytical and multidisciplinary process which requires human resources with highly specialized training and a wide breadth of information. The government should consider implementing postgraduate training in HTA, locally and internationally, and to ensure a critical mass of HTA researchers to conduct studies and meet the growing demand for HTA. HTA capacity should be developed in research organizations as well as in decision-making bodies and other relevant stakeholders who use HTA. Fostering HTA training programs at local academic institutions is one of the most significant factors that should be considered as part of HTA institutionalization.

## Conclusions

China has a complex and dynamic pharmaceutical system undergoing significant reforms as part of the national health care reforms. The complicated and interdependent problems interact in a way that leads to significant system problems, which create dual challenges that both the developing country and the developed countries are facing. To further promote access to medicines, China should speed up the re-assessment of the quality and efficacy of domestically produced generic medicines; coordinate various reforms of price determination, insurance payments, and procurement policies; address medicine shortages through comprehensive policies and legislation; establish specific mechanisms to achieve sustainable equitable access to expensive essential medicines with health technology assessment as a tool to ensure that policy and priority setting are created in a coherent and evidence-based way.

## Additional files

Additional file 1: Key pharmaceutical sector reform policies issued by the Chinese Communist Party Central Committee (CCPCC) & State Council (SC) (1997–2017). (DOC 31 kb)

Additional file 2: Outline of the key informants interview and focused group discussion. (DOC 28 kb)

## Abbreviations

GDP: Gross domestic product
HTA: Health technology assessment
LIMILCs: Low-income and middle-income countries
TPE: Total pharmaceutical expenditure
WHO: World Health Organization
