# Supplementary material for: A review of promoting access to medicines in China - problems and recommendations
Source: BMC Health Serv Res. 2018 Feb 20;18:125. doi: 10.1186/s12913-018-2875-6 (PMC5819167; doi:10.1186/s12913-018-2875-6)
Supplement: Supplementary file 1 — Key pharmaceutical sector reform policies issued by the Chinese Communist Party Central Committee (CCPCC) & State Council (SC) (1997–2017). (DOC 31 kb) [file 12913_2018_2875_MOESM1_ESM.doc]

1. **Key pharmaceutical sector reform policies issued by the Chinese Communist Party Central Committee (CCPCC) & State Council (SC) since (1997-2017)**

1997：CCPCC & SC. Decision of Health Reform and Development

2000：SC. Directive Opinions for Urban Health System Reform

2009：CCPCC & SC. Opinions for Deepening Health System Reform

2010：SC. Directive Opinions for establishment and regulation of procurement of essential medicines in the public primary care facilities

2010：SC. Opinions for Establishment and Strengthening Government Subsidy Mechanism for Primary Health Facilities

2011：SC. Key Tasks of the Five Major Reforms of the Health System Reform in 2011

2012：SC. Implementing Plan for Deepening Health System Reform during the 12^th^ Five-Year

2012：SC. The 12^th^ Five-Year Plan of Health Sector Development

2012：SC. The 12^th^ Five-Year Plan of National Medicines Safety

2012：SC. Opinions for Piloting Comprehensive Reforms in County Public Hospitals

2015：CCPCC & SC. Opinions for Pushing Pricing Mechanism Reform

2015：SC. Directive Opinions for Improving the Pooled Procurement of Medicines in Public Hospitals

2015：SC. Opinions for Reforming Evaluation System of Medicines and Medical Devices

2016：SC. Key Tasks for Deepening Health System Reform in 2016

2016：SC. Opinions for Implementation of the Dual Invoice System for Medicines Procurement in Public Health Facilities

2016：SC. Opinions for Re-evaluation of Quality & Efficacy of Generics

2017：SC. The 13^th^ Five-Year Plan for Deepening the Health System Reform

2017：SC. Opinions for Further Reform and Improve the Medicines Production, Distribution and Use

**Notes:** Reforms focused on initiatives in:

1) Containment of the rapid increase of pharmaceutical expenditures;

2) Formulation of appropriate medicines financing mechanisms including pricing of medicines, medical services, medical staff salary scales, reimbursement policies, and financing of public hospitals;

3) Secure stable and efficient supply and procurement of medicines; and

4) Sustainable universal access to essential medicine
