# Supplementary material for: A review of promoting access to medicines in China - problems and recommendations
Source: BMC Health Serv Res. 2018 Feb 20;18:125. doi: 10.1186/s12913-018-2875-6 (PMC5819167; doi:10.1186/s12913-018-2875-6)
Supplement: Supplementary file 2 — Outline of the key informants interview and focused group discussion. (DOC 28 kb) [file 12913_2018_2875_MOESM2_ESM.doc]

**2. Outline of the key informants interview and focused group discussion**

**Background**

In March 2016, the National Health and Family Planning Commission authorized the School of Public Health of Chinese Academy of Medical Sciences & Peking Union Medical College to carry out a comprehensive review of access to medicines in China since the 2009 national health system reform. The review aims to identify the problems impeding access to medicines, and to propose policy directions for formulation of the 13^th^ Five Year Plan for pharmaceutical sector development.

**Objective of the key informants interview and focused group discussion**

To learn the innovative reform initiatives in adequate and efficient medicines supply at affordable prices at local levels, and to learn the problems and demands in implementing relevant pharmaceutical sector reforms of multiple stakeholders.

**Targeted key informants**

1. Health reform departments（Development and Reform, Financing, Health, Insurance, and Industry Development）
2. Medicines regulatory authority
3. Provincial pooled procurement agency
4. Pharmaceutical industry and pharmaceutical association (production, wholesaler, retailer, logistics, electronic commerce)
5. Public hospital (pharmacy)

**Research questions**

In relating to the national pharmaceutical sector reform policies in the areas of medicines pricing, distribution, pooled procurement, shortages of essential medicines, separation dispensing from prescription, and access to high-priced medicines:

1. What innovative local pharmaceutical sector reform initiatives have been implemented in your province?
2. Any problems impeding the implementation of these reforms at local level?
3. Recommendations
